# Chondromesenchymal Hamartoma in Ectopic Thyroid Tissue in a Neonate

**DOI:** 10.1055/s-0039-1688803

**Published:** 2019-07-02

**Authors:** Agnes Szepesi, Zsolt Juhasz, Anna Kover, Bela Kajtar, Noemi Benedek, Endre Kalman, Tamas Kovesi, Marianna Imre, Peter Vajda

**Affiliations:** 1 Department of Pediatric Surgery, Petz Aladar County Teaching Hospital, Gyor, Hungary; 2 Department of Pediatrics, University of Pecs, Medical Center, Pecs, Hungary; 3 Department of Pathology, University of Pecs, Medical Center, Pecs, Hungary; 4 Division of Pediatric Anesthesiology, Department of Anesthesiology, University of Pecs, Medical Center, Pecs, Hungary; 5 Diagnostic Center of Pecs, Hungary

**Keywords:** chondromesenchymal, hamartoma, thyroid, ectopic

## Abstract

A full-term male neonate presented with a left sided cervical lump at the level of the thyroid gland. Magnetic resonance imaging (MRI) showed a benign heterogeneous solid mass with lobulated margins. The tumor underwent complete excision. Histology revealed the diagnosis of chondromesenchymal hamartoma in ectopic thyroid tissue.

## Introduction

Head and neck tumors are rare entities in neonates. They mainly consist of vascular malformations, other developmental lesions, and rarely other benign or malignant soft tissue tumors. 1

Cystic hygromas on the neck are the most common vascular malformations. They are classically found in the left posterior triangle of the neck. Large cystic hygromas can cause fetal hydrops and neonatal respiratory difficulties. 2 Ex utero intrapartum treatment (EXIT) procedure can be performed for urgent management of severe airway obstructions caused by the detected giant neck mass. 3

We report on a neonate with cervical mass which turned out to be a hamartoma arising from ectopic thyroid tissue.

## Case Report

A full-term 1-day-old male neonate with a birth weight of 3,420 g was admitted to our department with a left sided cervical mass. The patient had a history of fetal tachycardia and maternal fever during the pregnancy. However, the cervical lesion was not detected on antenatal ultrasounds.

**Fig. 1:**
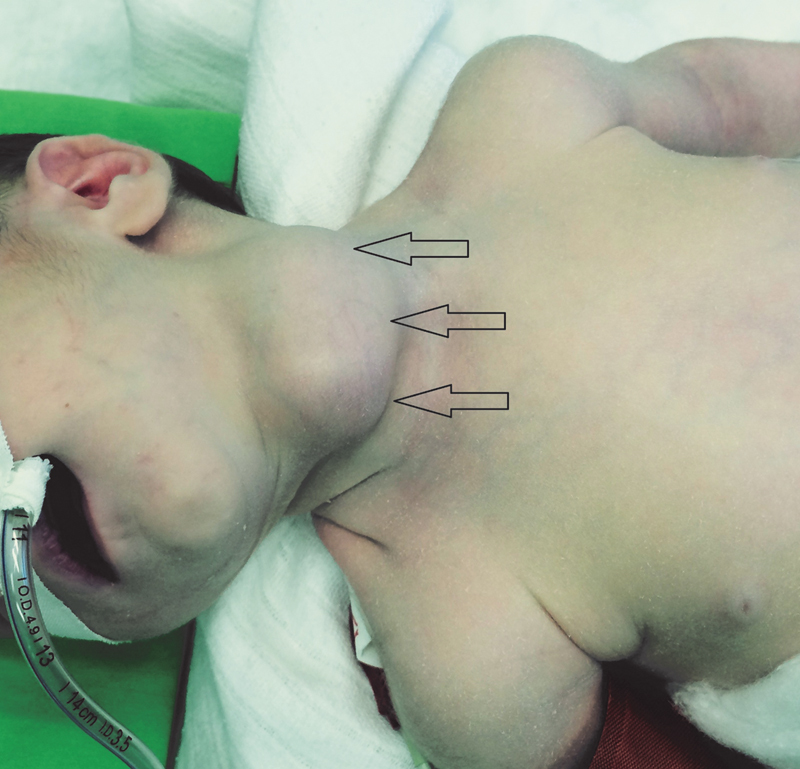
Solid mass on the neck extending from the left side to the midline (arrows).

On physical examination, the patient had a painless mobile mass on the left side of the neck at the level of the thyroid gland, 3 to 4 cm in diameter ( Fig. 1 ).

Otherwise no abnormality was detected.

**Fig. 2:**
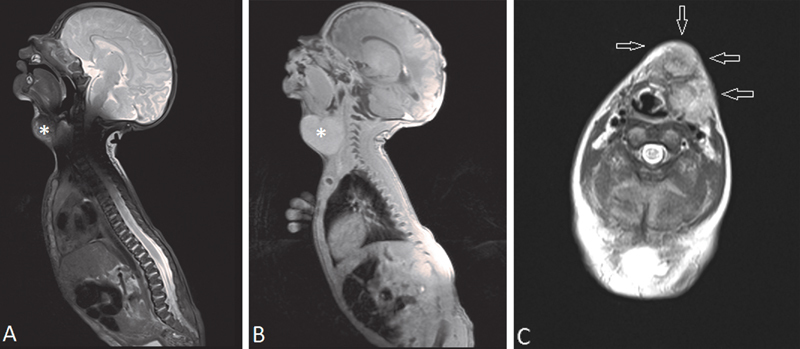
( **A, B** ) Sagittal T2 ( **A** ) and T1-weighted ( **B** ), MRI images, showing a lobulated tumor (asterisks) at the level of the thyroid gland, extending from the left side to the midline. ( **C** ) Axial, T2-weighted MRI demonstrates a tumor (arrows) on the left side of the neck at the level of the thyroid gland, dislocating the hypopharynx, larynx, trachea, and major vessels.

On magnetic resonance imaging (MRI) a large mass was seen in the soft tissue of the neck extending from the left side to the midline. The heterogeneous enhancing lobulated tumor slightly dislocated the hypopharynx, larynx, and trachea, causing mild tracheal dislocation and compression. The major vessels of the neck were also mildly dislocated. The morphology of the mass on MRI was not specific for any type of tumor. The thyroid gland was of normal size, shape, and structure. No pathologic lymph nodes or signs of invasion of surrounding tissues were detected ( Fig. 2A–C ).

**Fig. 3:**
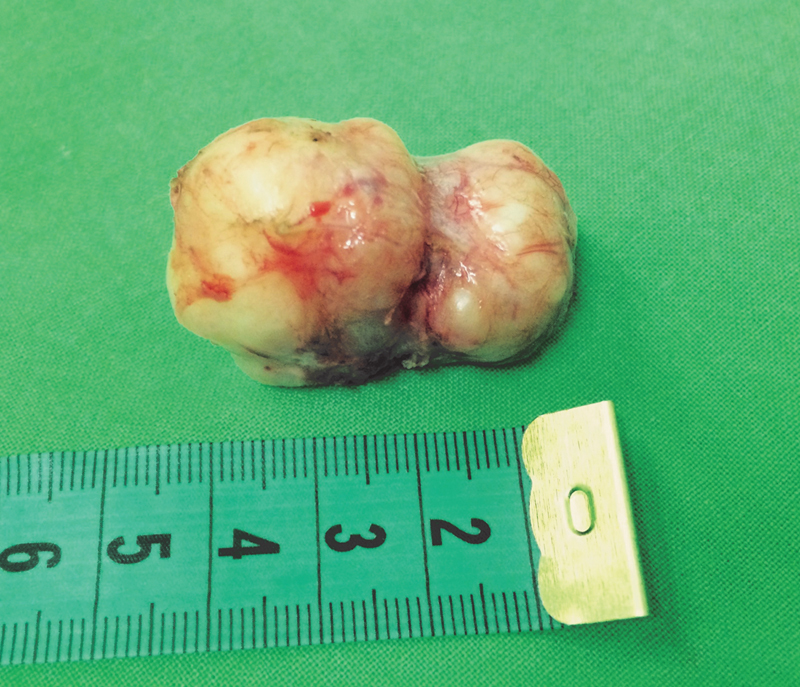
The completely removed tumor.

On the 10th day of life, the tumor underwent complete removal via Kocher's incision. The excision of the tumor was straightforward; the tumor was excised within its capsule without any injuries of the cervical structures ( Fig. 3 ).

The perioperative period was uneventful. No recurrence was detected during the 6-month follow-up.

## Pathological Finding

On gross examination, the 1 cm mass was lobulated, firm in consistency, gray–white in color, and well demarcated.

**Fig. 4:**
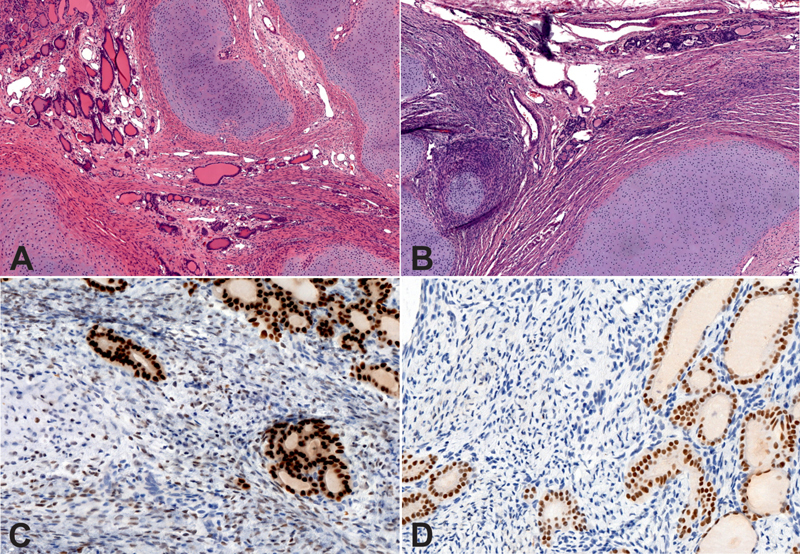
Histology of the specimen. ( **A, B** ) Large lobules of hyaline cartilage are visible separated by spindle cells and scattered thyroid follicles (×5 magnification, hematoxylin-eosin staining). ( **C** ) Thyroid follicles and spindle cells show strong and weak TTF1 staining, respectively (×20 magnification). ( **D** ) Only thyroid follicles show PAX8 staining (×20 magnification).

Microscopic examination revealed a cell-rich, immature mesenchymal tissue with spindle cells, and nodules of hypercellular hyaline cartilage without evidence of malignancy. Scarce mitotic activity (1/10 high power fields) was noted in the spindle cell component which demonstrated collagen fibers and myxoid areas. Focally thyroid follicles were noted within the mass. The spindle cells were S100, smooth muscle actin (SMA), neurofilament (NF), and paired box gene 8 (PAX8) all negative, but showed vimentin and weak thyroid transcription factor 1 (TTF1) staining. The thyroid follicles showed cytokeratin (CK), PAX8, and TTF1 expression ( Fig. 4A–D ).

The findings were consistent with a chondromesenchymal hamartoma arising in ectopic thyroid tissue.

## Discussion

The differential diagnosis of neck masses in children is based on clinical, pathological, and imaging characteristics. In childhood, most lesions are either congenital or inflammatory in origin and only a few neoplasms occur in the neck. The most common congenital developmental masses in the neck include thyroglossal duct cysts, branchial cysts, dermoid cysts, and vascular malformations. Inflammatory lesions can be a result of infectious lymphadenitis, reactive lymphadenopathy, or Kawasaki's disease. The differential diagnosis for neoplastic neck masses include benign tumors, such as haemangiomas, teratomas, plexiform neurofibromas, juvenile nasopharyngeal angiofibromas (JNA), and Langerhans cell histiocytosis (LCH), as well as malignant tumors, such as Hodgkin's lymphoma (HL) and non-Hodgkin's lymphomas (NHL), rhabdomyosarcoma (RMS), thyroid malignances, nasopharyngeal carcinomas (NPC), salivary gland tumors, neuroblastoma, and metastasis. 4 5 6

Teratoma is a subtype of germ cell tumors (GCT) derived from more than one of the three germinal layers. They are more common in the testes and ovaries, but can present in many different regions in the midline, including the sacral region, retroperitoneum, mediastinum, and brain. Mature teratomas are generally bening, immature teratomas in young children also tend to behave as benign tumors. In patients, older than 15 years, immature teratomas can manifest as highly devastating malignancies. 7

Hamartomas are benign congenital nonneoplastic abnormalities, but can be locally aggressive leading to complications. Ectopic thyroid tissue is another congenital developmental defect that may present as a tumor. The prevalence is 1 in 100,000 to 300,000; 90% of cases are localized to the base of the tongue. However, rare cases may appear in the cervical region resulting from abnormal migration and fusion of the lateral thyroid anlage. 8

Chondromesenchymal hamartomas are rare lesions, most occur nasally and are referred to as nasal chondromesenchymal hamartoma (NCMH). 9 Infants and children are predominantly involved but it occurs in adults as well. It presents mainly with sleep-disordered breathing due to nasal obstruction, feeding difficulties in infants, recurrent sinusitis, serous otitis media, epistaxis and watery rhinorrhea, decreased sense of smell, or frontal headache with no nasal symptoms. Orbital involvement and intracranial expansion may result in additional severe symptoms. In 2015, a systematic review was identified among 48 cases in the literature in adults and children but only in the nasal or cranial region. All patients underwent operative resection of the NCMH. Although NCMH is a benign lesion, malignant transformation has been reported in the literature. 4 9 10 11 12 13 14 15

The authors report the first case of chondromesenchymal hamartoma arising in ectopic thyroid tissue in neonate. Chondromesenchymal hamartomas usually arise from the nasal mucosa, a thyroid chondromesenchymal hamartoma is an exceedingly rare lesion, reported only in a few cases in the literature. 16 No cases of ectopic thyroid tissue developing chondromesenchymal hamartoma have been reported, so far. Considering the wide extension of the hamartoma, MRI was performed for preoperative planning and complete surgical excision. The specimen revealed no evidence of malignant transformation.

## Notes

New Insights and the Importance for the Pediatric Surgeon The authors report the first case of chondromesenchymal hamartoma arising in ectopic thyroid tissue in neonate.
